# Supracellular actomyosin assemblies: master coordinators of development

**DOI:** 10.1242/dev.204896

**Published:** 2025-08-26

**Authors:** Katja Röper

**Affiliations:** ^1^ Department of Physiology, Development and Neuroscience, University of Cambridge, Downing Street, Cambridge CB3 3DY, UK; ^2^ MRC Laboratory of Molecular Biology, Francis Crick Avenue, Cambridge Biomedical Campus, Cambridge CB2 0QH, UK

**Keywords:** Actomyosin, Cable, Morphogenesis, Supracellular, Epithelial cells

## Abstract

Most movement in biological systems is driven by assemblies of actomyosin, be it in the form of sarcomeres in muscles or as actomyosin networks in non-muscle cells. Actomyosin has several key functions within epithelial cells, the cells that will form most of the organs of an animal during development. One such function is to support cellular shape through an actomyosin cortex just underneath the plasma membrane. In addition, actomyosin accumulates apically at adherens and tight junctions, supporting cell-cell adhesion and epithelial tightness. Evidence over recent years has shown that apical actomyosin can also organise into ‘supracellular’ networks that seemingly span many cells. These large-scale assemblies either form interlinked networks of apical-medial actomyosin just underneath the free apical plasma membrane or form linear actomyosin cables at the level of adherens junctions. Both types of supracellular assemblies appear to be conserved across evolution, though were characterised in *Drosophila*. In this Review, I discuss the formation of these supracellular structures, the tissues in which they are known to function during development, their functional roles, and the remaining unknowns regarding their components and potential emergent properties.

## Teaser

**Summary:** Supracellular actomyosin assemblies in the form of apical-medial networks and junctional cables play key roles in tissue morphogenesis. This Review summarises their roles and characteristics, and the many open questions.

## Introduction

Tissues arise during development from simple precursors. These primordia become defined through transcriptional patterning, often with key transcription factors defining a particular organ fate. Transcriptional changes are upstream of the expression of so-called morphogenetic effectors that elicit the actual physical changes that sculpt tissues (Gilmour et al., 2017). An important consideration in the context of this Review is that transcriptional changes and downstream effectors work at the level of individual cells, but morphogenesis requires the concerted action of many cells across a tissue primordium. The transcriptional blueprint of a tissue therefore also needs to set in place factors that allow and control this tissue-wide coordination.

Morphogenetic effectors encompass cytoskeletal elements, such as actin and microtubules, and their respective motor proteins, which can directly induce physical changes and affect cell shape. These effectors also include actin and microtubule assembly and disassembly factors, as well as modulators of their activity and localisation (Sanchez-Corrales and Röper, 2018; Gillard and Röper, 2020; Röper, 2020). Much focus within the field of morphogenesis over the past 20 years has been on actin networks working with myosin (Murrell et al., 2015). In muscle cells, the stereotypical assembly of actin filaments and muscle myosin filaments into sarcomeric structures to allow coordinated contractility has been studied in much detail (Gautel and Djinovic-Carugo, 2016). In non-muscle cells, non-muscle myosin II can also form minifilaments that, when bound to actin networks, also function in a contractile fashion (Munjal and Lecuit, 2014; Koenderink and Paluch, 2018). Such actomyosin networks are key to supporting cell shape by forming the so-called actomyosin cortex underlying the plasma membrane. Modulation of the thickness, connectivity or contractility of this cortex provides a mechanism to change and adjust a given cell shape or drive cellular motility (Chugh and Paluch, 2018).

Tissue morphogenesis requires cellular changes and behaviour to be tightly coordinated. This is achieved in part through physical coupling of cells, and, in the case of epithelial cells, through cell-cell adhesion. Cadherins and immunoglobulin-like homophilic transmembrane receptors interact at apical adherens junctions, where their intracellular domains connect to the actomyosin cytoskeleton through intermediaries such as α-catenin, vinculin, ZO-1 and others (Röper, 2015; Mege and Ishiyama, 2017). This mechanical coupling means that changes in one cell are – to varying degrees – transmitted to neighbouring cells. Such direct coupling also allows for contractile changes in one cell to elicit mechano-chemical responses in neighbouring cells (Charras and Yap, 2018; Pinheiro and Bellaiche, 2018). Key players in the mechano-response of an epithelial cell are therefore also junctional cadherin-associated components, such as α-catenin and vinculin (Röper, 2015).

In epithelial cells, which form the majority of organs in all animals, junctional actomyosin is abundant and found in every cell (Cavey and Lecuit, 2009). In addition to supporting cell-cell adhesion, junctional actomyosin has been shown to underpin another core function of epithelial cells: that of providing a tight barrier. In epithelia, a paracellular and intramembrane diffusion barrier is assembled at tight junctions, located immediately apical to the adherens junctions in vertebrate cells (Citi, 2019). Reports indicate that the junctional actomyosin belt in epithelial cells in fact also stretches across part or all of the tight junctions and interacts with their transmembrane and membrane-associated components (Mangeol et al., 2024), and actomyosin-generated tension is crucial for proper epithelial tightness (Citi, 2019; Peterson et al., 2023). Whether this interaction affects any morphogenetic processes is still unclear.

In addition to the more ‘house-keeping’ functions of cortical actomyosin in supporting stable cell shape or driving cell shape changes, and in supporting adhesion and epithelial tightness, further intriguing networks have captured the attention of many labs over the past 20 years, assemblies we now call ‘supracellular’. These supracellular assemblies, built from actomyosin structures in individual cells and connected at cell-cell junctions, are coordinated between many neighbouring cells into large-scale arrangements. These assemblies become amplified to support or drive certain morphogenetic changes that require coordination among many neighbouring cells. In this Review, I illustrate the different types of supracellular assemblies, what recent studies have shown us about how they are built and positioned, and what we know about their specific functions.

## Types of supracellular actomyosin assemblies and where to find them

### Supracellular actomyosin cables

**Fig. 1.**
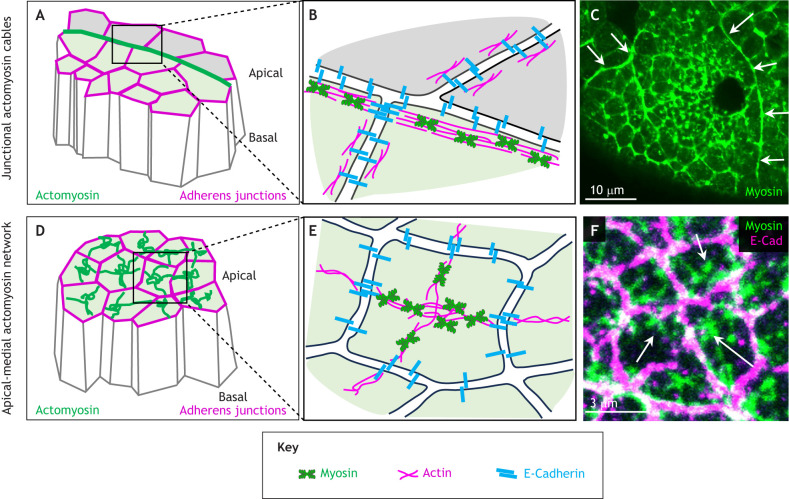
**Types of supracellular actomyosin assemblies.** (A) Schematic illustrating the apical and junction-associated position of a supracellular actomyosin cable (green) in epithelial cells. Apical and basal ends of the cells are indicated. Adherens junctions are labelled in magenta. (B) A more-detailed schematic of the positioning of a cable along bicellular junctions and how individual cable segments are connected across cell-cell junctions. Myosin is in green, actin is in magenta and E-Cadherin is in blue. (C) Example of a supracellular actomyosin cable (highlighted by white arrows) around the salivary gland placode in the *Drosophila* embryo. The frame is from a live time-lapse movie of non-muscle myosin light chain-GFP. Note the continuous appearance of the cable. Unpublished image prepared using the method described by Röper (2012). (D) Schematic illustrating the positioning and connectivity of supracellular apical-medial actomyosin networks (green). Apical and basal ends of the cells are indicated. Adherens junctions are labelled in magenta. (E) A more-detailed schematic of the apical-medial actomyosin assembly in a single cell of a supracellular network, with connections to spot adherens junctions where the apical-medial myosin connects between cells. Myosin is in green, actin is in magenta and E-Cadherin is in blue. (F) Higher magnification of the apical-medial actomyosin network (myosin-GFP is in green; the apical-medial network is highlighted by white arrows) and adherens junctions [marked by E-Cadherin (E-Cad) in pink] in the constricting area of the salivary gland placode. The myosin fibrils appear to be almost continuous across junctions. Unpublished image prepared using the method described by Booth et al. (2014).

**Fig. 2.**
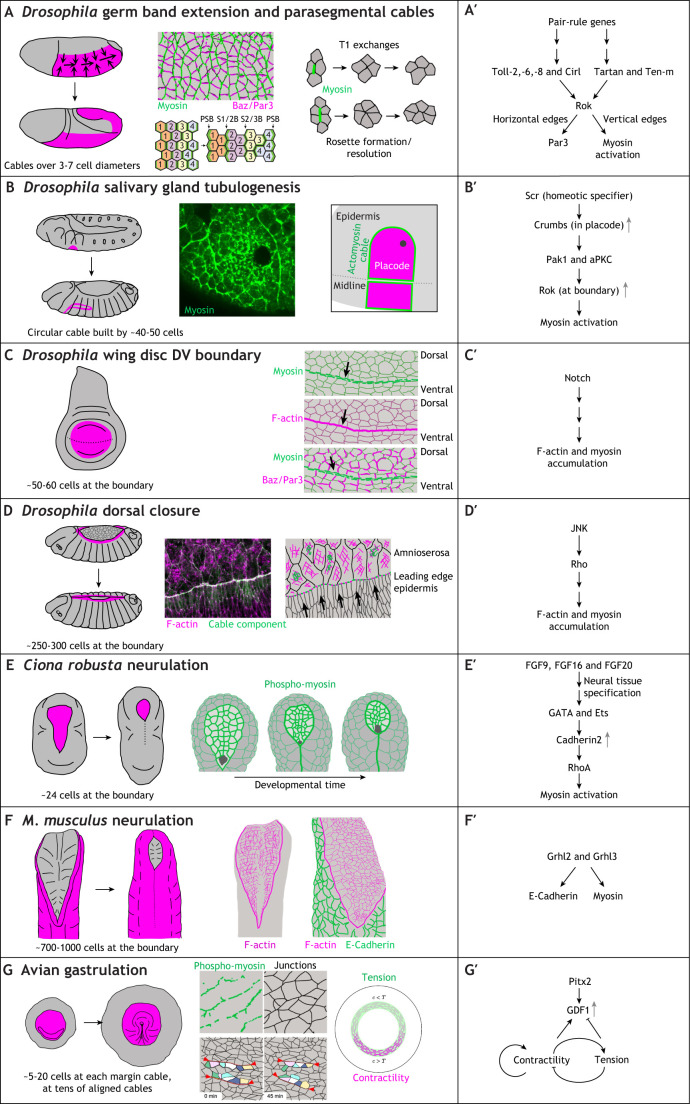
**Supracellular actomyosin cables in morphogenetic movements.** (A-G) For each process illustrated, the position of the tissues displaying the supracellular actomyosin cable(s) within them or at their boundary is highlighted in the schematic on the left in magenta, followed by images and/or schematics of the cables and their role in the process. (A′-G′) Genetic and signalling pathways identified that lead to assembly of the supracellular actomyosin cables, details of which are discussed in the main text. (A,A′) *Drosophila* germband extension is driven by DV-oriented dynamically constricting actomyosin cables that initiate both T1 exchanges as well as rosette formation/resolution exchanges (right; reproduced, with permission, from Vichas and Zallen, 2011). Middle top panel illustrates myosin cables (green) compared to complementary E-Cadherin and Bazooka/Par-3 enrichment. Schematic inspired by Fernandez-Gonzalez et al. (2009). Middle bottom panel illustrates how DV cables resolve over time into DV-oriented parasegmental boundary cables. Reproduced from Tetley et al. (2016), where it was published under a CC-BY 4.0 license. Cell rows (labelled 1 to 4), with DV-polarised myosin accumulations undergo neighbour exchanges to stretch out the germband, leading to strong myosin accumulation at the position of the forming parasegmental boundaries (PSBs) as well as lower-level myosin accumulation at boundaries between the forming wider stripes of similar cells (i.e. boundaries labelled 1/2 and 2/3). Cables in the germband are assembled downstream of pair-rule gene control and their positioning involves different sets of Tolls/LRRs (A′). (B,B′) The contractile cable at the boundary of the salivary gland placode in the *Drosophila* embryo is positioned by the anisotropic localisation of Crumbs in boundary cells, with Crumbs expression controlled by the most upstream homeotic factor specifying the tissue: Scr (B′). Images in B show the position of the cable at the boundary (myosin-GFP) using immunofluorescence (middle) and as a schematic with the placode in magenta, the cable in green and the invagination point in grey (right). Unpublished image prepared using the method described by Röper (2012). (C,C′) An actomyosin cable is formed at the DV compartment boundary in the *Drosophila* wing disc downstream of Notch signalling (C′). (C) Right panels illustrate the localisation of myosin (green, top), F-actin (magenta, middle) and myosin (green, bottom) versus Bazooka/Par-3 (magenta, bottom) in the wing disc. Schematic inspired by Major and Irvine (2006) (D,D′) During dorsal closure, an actomyosin cable forms at the leading edge of the epidermis that is moving dorsal-wards, with the assembly of the cable initiated by JNK signalling (D′). The cable is formed in the leading edge epidermal cells, as illustrated in the middle panel in D, with F-actin in magenta and a cable component in green. Unpublished image prepared using the method described by Ashour et al. (2023). (E,E′) During *Ciona robusta* neurulation, the anisotropic localisation of Cadherin2 (downstream of GATA, Ets transcription factors) drives anisotropy of RhoA and therefore myosin activation (E′). Formation of an actomyosin cable at the neuroectoderm-surface ectoderm boundary is illustrated in E (right) showing where activated phospho-myosin localises. Schematic inspired by Hashimoto and Munro (2019) (F,F′) During mouse neurulation, an actomyosin cable is found at the neuroectoderm-surface ectoderm boundary, induced by ectodermal expression of Grhl2 and Grhl3 (F′). (F, right) The localisation of F-actin (magenta) and F-actin in comparison to E-Cadherin (green) at the ectoderm-neuroectoderm boundary. Schematic inspired by Galea et al. (2017). (G,G′) During avian gastrulation, in the disc-shaped embryo, a network of shorter circumferential actomyosin cables accumulates at the boundary of the embryo proper and the extraembryonic tissue (middle panels in G; phospho-myosin in green, junctions in black and the contraction of the cables illustrated by frames of a movie). Inspired by Saadaoui et al. (2020) (G′) A feedback loop involving contractility/tension and expression of the TGFβ-like factor GDF1 is involved in the set-up and maintenance of these actomyosin cables. Right schematic in G reproduced from Caldarelli et al. (2024), where it was published under a CC-BY 4.0 license.

**Fig. 3.**
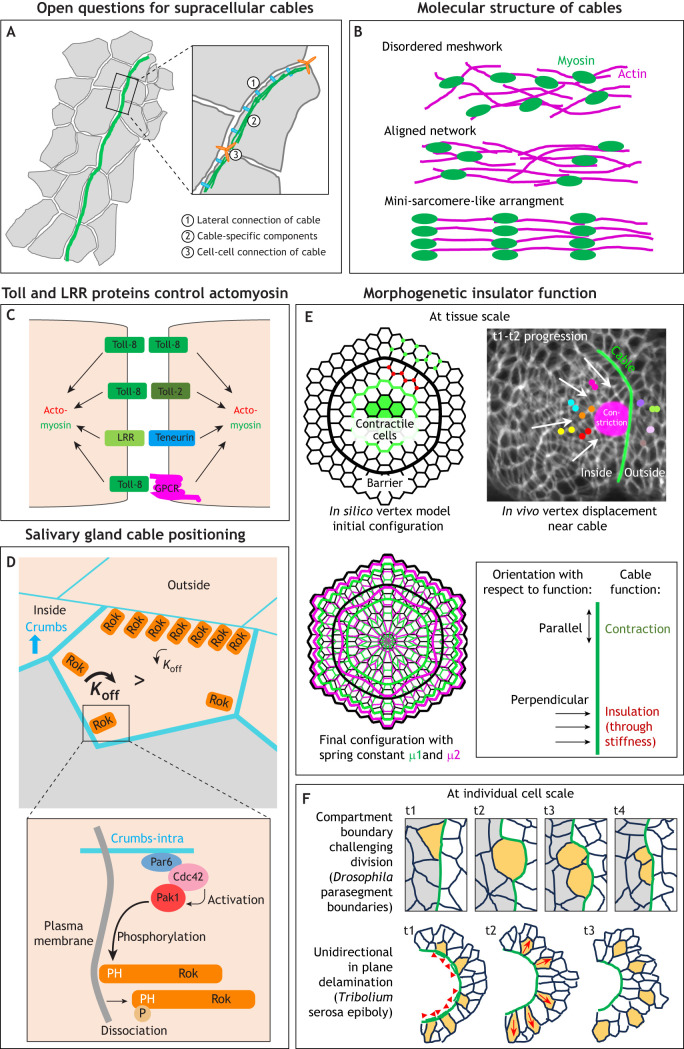
**Regulation, functions and unknowns of supracellular actomyosin cables.** (A) Open questions about the organisation of supracellular actomyosin cables include: is there any lateral connection to the bicellular junction (1); are there cable-specific components (2); and at what type of junctions are the individual segments of cables connected between cells (3). (B) Actomyosin cables could display increased molecular order to execute their function: most junctional actomyosin is presumed to be a gel-like meshwork (top), whereas sarcomeric actomyosin in muscles is organised into a near crystalline array (bottom). The presence of muscle-components such as Zasp52 in cables could indicate an intermediate state of an aligned network in cables (middle). (C) Striped expression of Toll receptors and other leucine-rich repeat receptors (LRRs) in the *Drosophila* embryo can control junctional actomyosin accumulation, including where cables form. Potential interactions include homophilic and heterophilic Toll-Toll binding, Toll-GPCR binding and Toll/LRR-Teneurin binding. The downstream mechanisms linking the receptors to actomyosin remain to be explored. (D) At the boundary of the salivary gland, the anisotropic localisation of Crumbs affects Rok residence time (top), leading to Rok accumulation at the boundary of the placode. Crumbs intracellularly recruits Pak1 (bottom), which can phosphorylate the PH-domain of Rok, leading to its membrane dissociation. Adapted, with permission, from Sidor et al. (2020), where it was published under a CC-BY 4.0 license. (E) The cable at the salivary gland placode boundary can act as a morphogenetic mechanical insulator, leading to the pull of the constriction of the invagination spreading to only inside cell nodes, but not to outside nodes beyond the cable. This is shown in the schematic of a time overlay (top right; cable in green, area of constriction in magenta; coloured dots mark cell nodes on the inside and outside of the placode). Two timepoints (t1 and t2) of a time-lapse movie of labeled membranes in the placode have been superimposed. Arrows indicate movement of labelled nodes on the inside. Reproduced from Ashour et al. (2023), where the image was published under a CC-BY 4.0 license. *In silico* vertex modelling suggests that mechanical insulation could be a common function of cables, with a higher spring constant of the cable (the barrier in the model) leading to better insulation. Schematics show the initial set-up of the model with a constricting zone in green in the centre, a barrier in black representing the cable, and cell nodes on the inside and outside, the motion of which is analysed. The lower left schematic shows the initial configuration (black) and two final configurations, with different spring constants of the cable implemented (green and magenta). Reproduced, with permission from Ashour et al. (2023), where it was published under a CC-BY 4.0 license. The contraction function and insulation function of the cable are distinguished by their orientation with respect to the cable extension: parallel and perpendicular, respectively. (F) At the individual cell scale, the contractility of cables can function as a physical boundary to correct challenges to compartment boundaries caused by dividing cells (top panels, dividing cell in yellow, parasegmental cable in green). Schematic inspired by Monier et al. (2010). Alternatively, cable contractility can function to direct in plane delamination of cells from a contracting cable, as in *Tribolium* serosa epiboly (bottom panels, cells moving away from the cable in green are marked in yellow). The lower schematic is adapted from Jain et al. (2020), where it was published under a CC-BY 4.0 license.

The first supracellular assembly of actomyosin was observed not in a developmental context but during embryonic wound healing (Fig. 1A-C; Martin and Lewis, 1992). In both the vertebrate and invertebrate embryonic epidermis, a supracellular actomyosin cable, historically described as actin cable or actin purse string, assembles at the wound edge during epidermal healing, highly enriched in actomyosin and under increased tension compared to neighbouring junctions (Wood et al., 2002; Kobb et al., 2017). Similar structures were soon observed during unperturbed morphogenetic processes in embryogenesis, such as dorsal closure in the fly embryo, where a prominent actomyosin cable assembles at the leading edge of an advancing epithelial front of cells (Hayes and Solon, 2017). Actomyosin cables run along bi-cellular apical junctions, and individual subcellular segments are connected between neighbouring cells at bicellular junctions – likely through cadherin-based junctional complexes similar to general cell-cell adherens junctions (Fig. 1A,B) – even if in many cases, using confocal or similar light microscopy, the cables appear continuous across many cells (Fig. 1C). The list of processes and places where supracellular actomyosin cables have now been observed during morphogenesis is long and is still growing. It includes germband extension and parasegmental boundaries, dorsal closure, salivary gland tubulogenesis, the formation of posterior spiracles and dorsal appendages, and the wing discs in *Drosophila*, as well as at rhombomeres in zebrafish and during neurulation in *Ciona* and mouse (Figs 2 and 3). These are discussed in detail in this Review. Across the different processes in which they are observed, supracellular actomyosin cables differ in size, lifetime, dynamics and known constituent components, so it is currently unclear whether they serve a single unifying function or many parallel functions (Röper, 2013). The ‘assembly instructions’ for several cases of supracellular actomyosin cables have been elucidated over the past decade, and common themes have emerged that I will discuss below. In the case of some actomyosin cables, the term ‘supracellular’ reflects the observation that, even with advanced super-resolution imaging, the actomyosin structure appears smooth and continuous within cells and tightly coordinated at cell-cell junctions (Röper, 2012).

### Supracellular apical-medial actomyosin networks

**Fig. 4.**
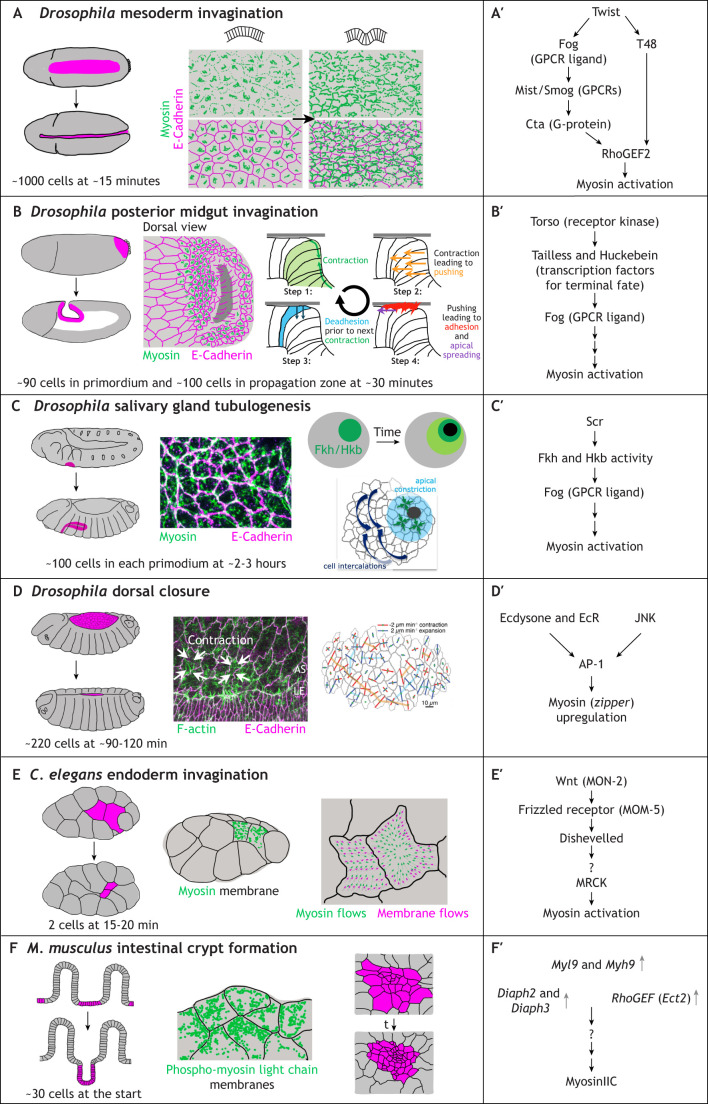
**Supracellular apical-medial myosin networks in cell constriction and tissue invagination.** (A-F) For each process illustrated, the position of the tissue displaying the apical-medial actomyosin network is highlighted in the schematics on the left in magenta, followed by images or schematics of the networks and their role in the process. (A′-F′) Genetic and signalling pathways identified that lead to assembly of the supracellular actomyosin cables, details of which are discussed in the main text. (A,A′) *Drosophila* mesoderm invagination during gastrulation leads to the inward bending of the mesoderm. Mesodermal cells accumulate a dense interlinked network of apical-medial actomyosin, leading to apical constriction and cell wedging that drives tissue bending (A, right; schematics inspired by Martin et al., 2009). (A′) Mesoderm specification and invagination is under the overall control of Twist, leading to a cascade of GPCR, RhoGEF2 and myosin activation. (B,B′) *Drosophila* posterior midgut invagination progresses as a wave that sweeps towards the posterior end of the embryo, with the initial invaginating zone, which is induced by Tailless and Hkb expression (B′), showing an extensive apical-medial actomyosin network (B, middle; myosin in green and cell junctions in magenta; inspired by Bailles et al., 2019) (B, right; inspired by Bailles et al., 2019) This network contracts (1), pushing more-posterior cells upwards (2) where they spread over the apically localised vitelline membrane (3) and adhere to it (4), before further contractility leads to de-adhesion (5). (C,C′) *Drosophila* salivary gland tube formation, commencing from a flat epithelial primordium of 100 cells leading to a narrow tube at the end of embryogenesis, shows a dynamic network of apical-medial actomyosin that drives the invagination (C, middle panel; cells near the invagination point, myosin in green, E-Cadherin in magenta). Unpublished image prepared using the method described by Booth et al. (2014). (C, right; C′) The medial network is activated downstream of radially expanding Fkh and/or Hkb transcription factor activity (green circles in C, right). Directional cell intercalations move new cells towards the apical constriction area (light blue). (C, right) Reproduced from Sanchez-Corrales and Röper, 2018, where it was published under a CC-BY 4.0 license. (C′) The homeotic transcription factor Scr activates Fkh and Hkb transcription, which in turn drive expression of the GPCR ligand Fog, ultimately leading to myosin activation. (D,D′) During dorsal closure in the *Drosophila* embryo, the constriction of amnioserosa cells is driven by an extensive network of apical-medial actomyosin [D, middle; F-actin is in green and E-Cadherin is in magenta; the leading edge epidermal cells (LE) and amnioserosa cells (AS) are indicated; white arrows illustrate the constriction of cells]. Reproduced from Ashour et al. (2023), where it was published under a CC-BY 4.0 license. (D, right) Constriction of amnioserosa cells can be coupled between neighbours (red lines indicate contraction, blue lines expansion, a brown line links a row of neighbours all in a contraction phase). Reproduced from Blanchard et al. (2010), where it was published under a CC-BY 4.0 license. (D′) Myosin upregulation in the amnioserosa is induced by the transcription factor AP-1 that is controlled by both the hormone ecdysone and JNK signalling. (E,E′) During the internalisation of the two endodermal cells in the early *C. elegans* embryo, myosin accumulates at the apical surface of these cells (E, middle; myosin in green, membranes in black; inspired by Roh-Johnson et al., 2012). (E, right) Dynamic analysis reveals strong apical-medial myosin flows, linked by a clutch mechanism to drive membrane movement (green arrows show myosin flows; magenta arrows show membrane movement; schmatic inspired by Roh-Johnson et al., 2012). (E′) The myosin activation in endodermal cells is controlled by Wnt (MON-2). (F,F′) The formation of intestinal crypts in the mouse intestine (left) follows on from the formation of villi and involves the apical constriction of intervillar cells. (F, right) Two timepoints that display active myosin (magenta) across their apical surfaces; inspired by Sumigray et al. (2018). (F, middle) The constricting cells display high levels of apical phospho-myosin light chain, green; membranes are shown in black; inspired by Sumigray et al. (2018). (F′) The regulation of the process is unknown, apart from the observed increased mRNA levels of myosin, as well as regulators of actin dynamics.

**Fig. 5.**
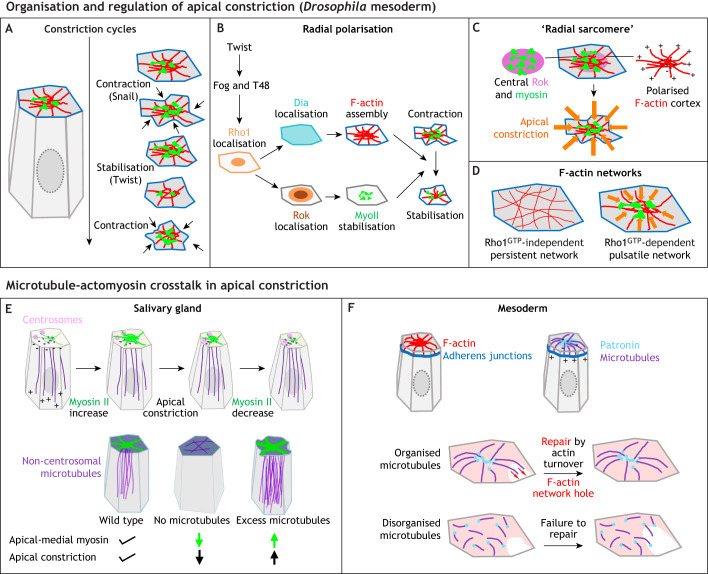
**Molecular pathways impinging on apical-medial actomyosin assemblies.** (A-D) Organisation and regulation of apical constriction in the *Drosophila* mesoderm. (A) Apical-medial myosin undergoes pulsatile increases in intensity and condensation that drive repeated cycles of constriction, with contraction being affected in *snail* mutants and the stabilisation of a new apical area failing in *twist* mutants, indicating different roles for Twist and Snail in controlling the process. Inspired by Martin et al. (2009) (B) The apical-medial network displays radial polarisation in each cell, with different pools of Rho1 in medial and junctional positions controlling Rok and Dia localisation, respectively, and thereby affecting the F-actin network and the myosin working on this network, to control both contraction and stabilisation. Inspired by Mason et al. (2013) (C) The radial polarisation is evident in a ‘radial sarcomere’ arrangement of F-actin with barbed (plus) ends at the periphery and pointed (minus) ends in the centre, where they colocalise with Rok and myosin, leading to efficient apical contraction. Schematic inspired by Coravos and Martin (2016) (D) The F-actin network consists of two interlaced parts: a persistent network that is Rho1-GTP independent (left); and a pulsatile network that is dependent on Rho1-GTP (right). (E,F) Microtubule-actomyosin crosstalk in apical constriction in the *Drosophila* embryo. Schematic inspired by Dehapiot et al. (2020) (E) During apical constriction in the *Drosophila* salivary gland placode, the apical-medial actomyosin network depends on the underlying non-centrosomal longitudinal microtubule network for its assembly and function (top row: centrosomes in pink, microtubules in purple, actomyosin in green; microtubule minus and plus ends are indicated; inspired by Booth et al., 2014). Adapted from Gillard et al. (2021), where it was published under a CC-BY 4.0 license. There appears to be a fine balance in the amount of microtubules required to achieve the correct level of constriction, with loss of microtubules abolishing apical-medial myosin and constriction, and excessive microtubule nucleation increasing apical-medial myosin and constriction (bottom row: microtubules are in purple and myosin is in green). Adapted from Gillard et al. (2021), where it was published under a CC-BY 4.0 license. (F) During *Drosophila* mesoderm invagination, microtubules are also key to correct apical-medial actomyosin function, with a radially polarised apical microtubule network important to guide the repair of the apical F-actin network. The top schematic illustrates the microtubule and actin arrangement (adherens junctions in dark blue, F-actin in red, microtubules in purple and the minus-end binding protein Patronin in light blue). Adapted from Ko et al. (2019), where it was published under a CC-BY 4.0 license. Bottom schematic illustrates the effect of microtubule organisation or its loss on the ability to repair the actin network (microtubules in purple, Patronin in blue, F-actin network in red). Adapted from Ko et al. (2019), where it was published under a CC-BY 4.0 license.

The second type of supracellular actomyosin assembly only entered the stage in 2009: apical-medial actomyosin networks (Fig. 1D,E; Bertet et al., 2009; Martin et al., 2009; Solon et al., 2009). Initially, these networks were not fully understood to be supracellular; this characteristic was only uncovered over time. Supracellular apical-medial actomyosin networks, as the name suggests, assemble as dynamic actomyosin densities in the apical-medial region of epithelial cells, with connections to spot adherens junctions at the periphery of the cells, where they connect between neighbouring cells (Fig. 1D-F). These apical-medial myosin networks, rather than the junctional actomyosin belt, have emerged as the main drivers of apical constriction of epithelial cells, a process underlying morphogenetic events such as tissue bending or invagination (Martin, 2010). Increased levels of myosin had already been found in the apical region of invaginating or bending epithelial cells two decades ago (Young et al., 1991), but the prevalent junctional actomyosin ‘belt’ in epithelial cells was assumed to be key to this. Apical-medial actomyosin networks are highly dynamic, displaying pulsatile, cyclical increases and decreases in intensity when imaged that correlate with the rate of apical area constriction (Martin et al., 2009; Miao and Blankenship, 2020). This pulsatile behaviour reflects increases and decreases in the amount and density of actomyosin within the apical-medial region. After the initial observation that apical-medial actomyosin activity is responsible for mesoderm invagination in *Drosophila* (Martin et al., 2009), such networks have been identified to underlie many epithelial morphogenetic processes. This includes posterior midgut invagination, salivary gland morphogenesis and amnioserosa shrinkage in *Drosophila*; endoderm internalisation in *C. elegans*; and intestinal crypt formation in mouse (Figs 4 and 5). I discuss these in detail this Review. Despite their dynamic nature, individual apical-medial actomyosin pools in cells are connected through actin filaments to adherens junctions, through which the pool of one cell is tied to the much wider network spanning many cells (Fig. 1D-F; Martin et al., 2010).

One poorly understood aspect of large-scale actomyosin assemblies, be it apical-medial networks or cables, is whether there are specific functions or effects that either scale with the size or the supracellularity or that might emerge only in large-scale networks. Do we find different functionalities when comparing multicellular and supracellular assemblies? Starting from supracellular actomyosin cables and moving to apical-medial supracellular networks, I will now discuss recent advances in our understanding of the assembly and regulation of these structures, and our current understanding of their roles.

## Supracellular cables: assembly, regulation and functions

### *Drosophila* germband extension and parasegmental cables

Germband extension is a key process in *Drosophila* gastrulation that leads to an approximate doubling of the length of the anterior-posterior (AP) axis of the embryo whilst halving its width (Fig. 2A). Pioneering work over the past two decades has revealed that convergence and extension movements of epidermal cells drive the elongation, and are powered and organised by dynamic supracellular actomyosin cables (for a review, see Pare and Zallen, 2020). Actomyosin is enriched in vertical cell boundaries, leading to their contraction and subsequent pulling of groups of four or more cells into a vertex or rosette, with both then resolving perpendicularly, thereby reducing the number of cells along the dorso-ventral (DV) axis and increasing the number in the AP axis. The preferential myosin accumulation in vertical junctions is driven by polarised localisation of Rho kinase (Rok) at these junctions (Simoes et al., 2010). The upstream cascade linking to Rho/Rok in this case is not yet understood, but it is downstream of the segmental patterning of pair-rule genes, leading to the placement of two downstream systems that affect actomyosin junctional accumulation. One system involves striped expression of leucine-rich repeat (LRR)-containing Toll receptors (Pare et al., 2014); the other involves striped localisation of another LRR receptor, Tartan, and its binding partner the teneurin Ten-m (Fig. 2A′) (Pare et al., 2019; Sharrock et al., 2022). The DV-oriented enrichment of myosin resolves over time into strong accumulation at parasegmental boundaries at stage 8-10 (Tetley et al., 2016). These boundaries act to restrict differently fated cells within their correct compartments, in particular when these boundaries are challenged by cell division. Parasegmental cables have been shown to maintain daughter cells within their correct compartment (Monier et al., 2010).

### *Drosophila* salivary gland tubulogenesis

The salivary glands form halfway through embryogenesis from flat bilateral epidermal primordia, so-called placodes of about 100 cells each, within parasegment 2 of the *Drosophila* embryo (Fig. 2B; Panzer et al., 1992; Andrew et al., 2000; Girdler and Röper, 2014; Sidor and Röper, 2016). At the time of specification (mid to late stage 10), the earlier parasegmental cables have all disappeared, apart from the ventral part of the two parasegmental cables framing parasegment 2. These then connect up over the dorsal side of the placode, and are ventrally linked to cables running along the length of the ventral midline of the embryo (Röper, 2012), thereby forming an overall circumferential actomyosin cable positioned at the boundary of the salivary gland placode. The physical position of this cable is determined by increased levels of the apical polarity determinant Crumbs in the placode. Crumbs is a transmembrane protein that can undergo homophilic interactions between neighbouring cells. The difference in Crumbs levels between placodal and surrounding epidermal cells then leads to the anisotropic localisation of Crumbs only in placodal cells located right at the boundary of the placode (Fig. 2B′) (Röper, 2012; Thompson et al., 2013). Intracellularly, Crumbs recruits kinases that can phosphorylate the membrane-binding pleckstrin homology (PH) domain of Rok, leading to its membrane dissociation (Fig. 2B′). The residence time of Rok is therefore higher at the Crumbs-depleted boundary membrane, driving myosin accumulation and cable assembly (Sidor et al., 2020). The function of the placodal cable is not completely understood, but it contracts concomitantly with the invagination of placodal cells into the embryo to form the tube of the salivary gland. Thus, the contraction might help the orchestrated movement of placodal cells known to be key to correct invagination (Sanchez-Corrales et al., 2018, 2021). In addition, the placodal cable also functions as a mechanical insulator, preventing the large-scale morphogenetic changes within the placode from unduly influencing the surrounding tissue (Ashour et al., 2023). Supracellular cables surrounding tube organ primordia have also been reported in *Drosophila* embryonic posterior spiracles and the dorsal appendages of the forming egg (Simoes et al., 2006; Osterfield et al., 2013), exhibiting very similar topology involving a near circular placode and a circumferential cable.

### *Drosophila* wing disc dorsoventral boundary

The accumulation of actomyosin or full-scale cables at fate boundaries is a common theme and also applies in the *Drosophila* wing disc, where myosin accumulation at the DV boundary into a cable-like structure was observed nearly 20 years ago (Fig. 2C; Major and Irvine, 2005). The establishment of the DV boundary itself is regulated by Notch signalling, which also drives the accumulation of actomyosin at the boundary (Fig. 2C′) in a complementary pattern to Par-3/Bazooka accumulation in the boundary cells. This is reminiscent of a similar complementary accumulation in the cables driving germband extension in the embryo (Major and Irvine, 2006; Simoes et al., 2010). Clones of cells in wing discs with altered Notch signalling are rounded and accumulate actomyosin at their boundaries (Major and Irvine, 2006). This might mimic DV boundary formation, but might also be a more general feature and response of wing discs, which have been shown to respond to ectopically induced clonal fate borders with myosin accumulation at these ectopic boundaries (Bielmeier et al., 2016). Higher interfacial tension is also observed at the AP compartment boundary in the wing disc and the AP boundary in the pupal abdomen in histoblast nests that form the precursors of the adult epidermis, though in these cases there is no easily recognisable myosin accumulation into anything cable-like, so the molecular source of the increased tension in unclear (Wang et al., 2023).

### *Drosophila* dorsal closure

One of the most prominent and well-studied actomyosin cables is found during the process of dorsal closure in the fly embryo (Fig. 2D). In this process, the two halves of the embryonic dorsal epidermis move together to close over the extra-embryonic tissue called the amnioserosa that, up to this point, covers the dorsal part of the embryo. Whilst the epidermal cells advance in a united front towards the dorsal midline, a thick accumulation of actomyosin forms at the leading edge of the dorsal-most epidermal cells. The advancement of the epithelial sheets is downstream of JNK/Dpp signalling (Riesgo-Escovar and Hafen, 1997; Reed et al., 2001), with the small GTPase Rho playing a key role in the regulation of the cytoskeleton at the leading edge (Fig. 2D′) (Jacinto et al., 2002). Assessing the role of the cable in dorsal closure has not been straightforward, as multiple processes play a part in closure: the movement of the epidermis, the contraction of the amnioserosa and, at later stages of closure, the interactions between the two epidermal sheets as the embryo closes. Leading edge epidermal cells interact at the canthi of the hole through long filopodia, driving a zipping-up process that also drives segment matching between the lateral sides (for a review, Hayes and Solon, 2017). Laser ablations show that the cable is under tension, but genetic inhibition of myosin activity in either the amnioserosa or the leading edge epidermal cells suggests that actomyosin cable tension is not the major driver of closure (Pasakarnis et al., 2016). Rather, the cable assists correct closure by enabling a united front of cells to move dorsalwards, whereas without proper cable function, the forming dorsal midline is not straight (Ducuing and Vincent, 2016). Closure events are common in developing embryos; during *Tribolium castaneum* development*,* the extra-embryonic serosa closes around the forming embryo proper in a process of epiboly. During this serosa closure, a supracellular actomyosin cable accumulates at the embryo-serosa boundary that is very similar to the *Drosophila* dorsal closure cable. This cable seems to allow ‘fluidisation’ of the serosa tissue near the cable, with cells delaminating away from the cable within the plane of the epithelium, thereby successively shrinking the cable circumference (Jain et al., 2020).

### *Ciona robusta* neurulation

Prominent supracellular actomyosin cables have also been observed more recently during neurulation events in different animal models. During *Ciona robusta* neurulation, a myosin cable assembles at the boundary of the neuroectoderm and surface ectoderm (Fig. 2E). Interfering with this cable leads to delays and failure in neural tube closure (Hashimoto and Munro, 2019). Neural induction in *Ciona* activates GATA and Ets transcription factors that drive increased expression of Cadherin2 in the neuroectodermal cells (Fig. 2E′) (Gainous et al., 2015). In a variation of the theme observed for the positioning of the cable at the salivary gland boundary in the fly embryo (Sidor et al., 2020), the increased levels of Cadherin2 in the neuroectoderm compared to the surrounding surface ectoderm leads to a strong anisotropic localisation of Cadherin2 only in cells at the boundary. Here, Cadherin2 recruits a RhoGAP (Gap21/23) that blocks Rho activation at the plasma membrane where Cadherin2 is high, leading to increased Rho activation at the boundary itself, where it promotes actomyosin accumulation (Fig. 2E′; Hashimoto and Munro, 2019).

### Mouse neurulation

In mouse neurulation, during the processes of both anterior and posterior neuropore closure, a large-scale actomyosin cable is found at the boundary of the neuroectoderm and surface ectoderm (Fig. 2F). Morphometric analysis and laser ablations of the cable suggest that its tension is important for closure (Galea et al., 2017; Maniou et al., 2021). Myosin enrichment at the surface ectoderm boundary appears to be regulated by the transcription factors Grainyhead-like 2/3 (Grhl2/Grhl3), with myosin lost from the boundary in mutants (Fig. 2F′) (Nikolopoulou et al., 2019). The balance of E-Cadherin being restricted in its expression to the surface ectoderm and N-Cadherin expression being restricted to the neuroectoderm is also affected in these mutants. The downstream effectors leading to myosin accumulation and activation at the boundary are still unidentified here.

### Avian gastrulation

During avian gastrulation, which starts in a flat, near-circular embryonic disc, actomyosin cables at the boundary of the embryo proper and extraembryonic tissue form a tensile ring (Fig. 2G). This circumferential assembly provides tension and contractility in different areas of the embryo to drive counter-rotational flows of cells that underlie avian gastrulation (Saadaoui et al., 2020; Caldarelli et al., 2024). A feedback loop between a TGFβ-family secreted factor, GDF1, and contractility and tension in the tissue underlies the self-organisation of the gastrulation movements (Caldarelli et al., 2024). Expression of GDF1 is induced by the transcription factor Pitx2 (Fig. 2G′, Torlopp et al., 2014).

## Common features of supracellular actomyosin cables and possible emergent properties

Although at lower resolution supracellular actomyosin cables look much alike, higher resolution can reveal further details. In some cables, the structure formed by myosin looks near continuous in imaging, even at cell-cell junctions joining cable segments between individual cells (salivary gland placode cable and germband extension cables). For other cables, myosin appears to form distinctly stronger accumulations in the centre of each intracellular cable segment (wing disc DV boundary and dorsal closure cables). It is still unclear how much the highly aligned arrangement of individual subcellular segments is a key aspect of supracellular cable function and could lead to emergent properties. For example, do neighbouring cable segments influence each other? This has been tested directly in a few cases using laser ablations of junctions that form part of a cable: during germband extension in *Drosophila*, laser ablation experiments have revealed that junctions that are part of DV-oriented multicellular cables (Fig. 2A) are under higher tension than other DV-oriented junctions, with junctions of longer cables displaying higher tension than those of shorter cables (Fernandez-Gonzalez et al., 2009). Furthermore, myosin turnover and accumulation in these cables is guided by tension. A similar dependency is found during wound repair in the *Drosophila* late embryonic epidermis, where fluorescence recovery after photobleaching (FRAP) analysis has demonstrated that myosin becomes stabilised in the forming actomyosin ‘purse-string’ cable surrounding a wound (Kobb et al., 2017). Furthermore, laser ablations within the cable revealed that the tension generated by the cable is necessary and sufficient to maintain and stabilise myosin at the wound edge, with a stronger effect on directly neighbouring junctions compared to more distant ones (Kobb et al., 2017). By contrast, during formation of the new AP boundary in the developing adult epidermis in the *Drosophila* pupa, myosin is only transiently enriched in the boundary, despite the boundary being tensed. Sequential laser ablations of nearby junctions at this boundary suggest that tension is generated cell autonomously (Wang et al., 2023). The difference between the pupal and embryonic systems could be due to the lack of an identifiable distinct actomyosin cable with locally strongly increased actomyosin levels in the pupal system. More studies addressing the entanglement between supracellularity of cables and their ability to generate tension or contraction across a variety of developmental processes are needed.

Many further questions remain with regard to supracellular cables, such as whether there are cable-specific components beyond actin, myosin and their more general regulators (e.g. Rok) that are not found in general junctional actomyosin (Fig. 3A). At least one component, the cytoplasmic protein Zasp52, shows some specificity for supracellular actomyosin cables (Ducuing and Vincent, 2016; Ashour et al., 2023). Zasp52 is found in most, but not all, actomyosin cables in the *Drosophila* embryo. It is also found within the Z-disc of muscle sarcomeres, where Zasp52 is required for proper sarcomere assembly via interactions with α-actinin (Jani and Schock, 2007). This dual specific localisation of Zasp52 suggests that the arrangement of actomyosin within supracellular cables might also be more ordered than in a cortical actomyosin meshwork (Fig. 3B). However, it is not clear whether this would mean only a slight alignment or a highly ordered array, such as that observed, for example, for junctional actomyosin in cochlear support cells in mouse (Ebrahim et al., 2013). It is also unknown whether adherens junctions that connect individual intracellular segments of supracellular cables are modified in any way to allow them to bear a higher tensional load (Fig. 3A). This could be achieved through specific junctional components or an amplification in the amount of some more-widespread junctional players. Finally, especially with regards to cables driving active junction constriction, it remains to be seen whether cables show any specific lateral junctional attachment as they run along bicellular junctions (Fig. 3A). Cables could be coupled to junctional adhesion complexes in a parallel configuration to the plasma membrane. Alternatively, they could be coupled to the plasma membrane in a way akin to how the cytokinetic actomyosin ring during cytokinesis is coupled to the plasma membrane during furrow ingression.

More advances have been made in determining how increased levels of junctional actomyosin or cables can be assembled at specific junctions or boundaries. A general theme has emerged whereby the anisotropic localisation of homophilic cell-cell adhesion receptors, such as cadherins, Echinoid (a nectin) and Crumbs, or of LRRs, such as Tolls/Toll-like receptors (TLRs), which is triggered by a sharp change in expression level of the receptor, can drive the junctional accumulation of myosin. In particular, in the early *Drosophila* epidermis, striped patterning of three different Tolls, as well as the LRR Tartan, the teneurin Ten-m and the GPCR Cirl is upstream of the selective accumulation of myosin at linked DV cell boundaries, leading to the formation of cables (Fig. 3C) (Pare et al., 2014; Tetley et al., 2016; Pare et al., 2019; Lavalou et al., 2021; Sharrock et al., 2022). The ability of TLRs to affect junctional actomyosin is conserved across evolution (Peterson et al., 2023). What links these membrane receptors to the control of actomyosin is less clear, but in the fly epidermis it appears to involve Src and PI3-kinase activity (Tamada et al., 2021). As already highlighted above, the cable at the salivary gland placode boundary in the fly embryo depends on the anisotropic localisation of Crumbs, which is again mediated by homophilic interactions and by the effect that recruitment of the kinases Pak1 and aPKC by the Crumbs intracellular tail has on Rok phosphorylation and residence time at the plasma membrane (Fig. 3D; Sidor et al., 2020). Anisotropic localisation of a Rho regulator at a boundary upstream of cable formation is also observed in *Ciona* neurulation (Fig. 2E; Hashimoto and Munro, 2019). The anisotropic patterning of Crumbs and complementary localisation of Crumbs and myosin is conserved in vertebrates, though whether this still plays a role in the assembly of supracellular actomyosin cables has not been determined (Francou et al., 2023). Interestingly, the same set of surface receptors appears to help to identify and possibly eliminate mis-specified or aberrant cells during development, through a mechanism of interface surveillance that involves the assembly of actomyosin cables at the interface of these mis-specified or aberrant groups of cells (Bielmeier et al., 2016; Fischer et al., 2024).

An exciting open question is whether the supracellularity of actomyosin cables elicits any emergent properties that individually working junctional myosin accumulations cannot provide, i.e. properties that go beyond the coupling of contractility. Live imaging and *in silico* modelling of the cable surrounding the salivary gland placode recently suggested that the whole cable functions as a morphogenetic insulator to prevent large-scale morphogenetic changes within the placode from unduly influencing the surrounding tissue, thereby preventing a spilling over of morphogenetic changes (Fig. 3E; Ashour et al., 2023). *In silico* vertex modelling has suggested that the stronger the spring constant of the cable, the stronger the insulation, likely because this increases the stiffness of the cable in an orientation perpendicular to its extension. The topology of a cable with respect to its function or functions is an important consideration here. For example, the placodal cable can support junction contraction to shrink the cable around the placode – this function is exerted in a parallel orientation to the cable extension. At the same time, it can support a mechanical insulator function that is exerted perpendicular to its extension, i.e. the stiffness of the cable prevents morphogenetic changes on one side of the cable from crossing over to the other side (Fig. 3E). This morphogenetic insulator function likely acts at tissue-scale, restricting larger tissue deformations and flows of cells to the correct organ primordia. At the individual cell scale, actomyosin cables also function as a physical boundary, for example by ensuring that cell divisions at a compartment boundary, such as the parasegmental boundary in the *Drosophila* embryonic epidermis, occur with daughter cells restricted to the correct compartment (Fig. 3F; Monier et al., 2010). In *Tribolium* during serosa epiboly, where an actomyosin cable drives the epiboly and then closure of the extraembryonic tissue, the boundary effect of the cable leads to selective delamination of cells within the plane of the epithelium away from the cable (Fig. 3F; Jain et al., 2020). Can we envisage other, as yet unproven, emergent properties? Circular supracellular actomyosin cables that are common during development could, for example, inject energy or noise into a morphogenetic system that could assist in jamming/unjamming transitions of epithelial primordia (Bi et al., 2015); however, for now, this remains speculative.

## Supracellular apical-medial networks: assembly, regulation and functions

The second type of supra-cellular assembly formed by actomyosin, supracellular apical-medial networks, is as prevalent in development as are supracellular cables; in fact, both are often also found in combination, providing the supracellular machines that sculpt tissues during morphogenesis.

### *Drosophila* mesoderm invagination

Mesoderm invagination as a key part of gastrulation in the *Drosophila* embryo has been studied for many years (Gheisari et al., 2020; Martin, 2020). During this process, about 1000 mesodermal cells invaginate into the embryo interior through the formation of a furrow or fold running from anterior to posterior, with the whole process of internalisation taking only about 15 min (Fig. 4A). In 2009, Martin and Wieschaus first described observing pulsatile apical area changes in mesoderm cells about to internalise. These pulsatile area changes correlated with intensity fluctuations and movements of myosin foci in the apical-medial regions, and not the junctional regions, of these cells (Martin et al., 2009). The upstream mesoderm-specifying transcription factors Snail and Twist control the constriction and accumulation of myosin and the stabilisation of a reduced apical area, respectively (Fig. 4A′). The medial myosin works in an apical-medial network of F-actin that is connected to apical cadherin-based junctions in distinct foci (Martin et al., 2010). Through these, the medial myosin is also interlinked between neighbouring cells, leading to the establishment of a highly interlinked and connected network with many nodes. The apical-medial network is established downstream of Twist through expression and secretion of the autocrine GPCR-ligand Fog (Folded gastrulation); upon binding to its GPCRs (Smog and Mist in the mesoderm), Gβ/γ activates Rho via RhoGEF2, and hence myosin (Fig. 4A′) (Seher et al., 2007; Manning et al., 2013; Manning and Rogers, 2014).

### *Drosophila* posterior midgut invagination

Immediately after the mesoderm has invaginated on the ventral side of the embryo, the invagination of the posterior midgut at the dorsal-posterior end of the fly embryo continues the process of gastrulation (Fig. 4B). Similar to the mesoderm, here, a dense network of apical-medial actomyosin accumulates in the primordium, eliciting apical constriction and infolding of an initial oval-shaped pocket (Bailles et al., 2019). This initial build-up of the apical-medial actomyosin network is transcriptionally controlled and, again, leads to expression of Fog, though here this is downstream of the transcription factors Huckebein (Hkb) and Tailless (Tll) (Fig. 4B′) (Casanova and Struhl, 1989; Weigel et al., 1990). The posterior-midgut invagination then spreads in a wave towards the posterior tip of the folded germband, leading to the consecutive invagination of further rows of cells that form the tube of the invaginating gut. Interestingly, the propagation of the wave is not dependent on transcription but, instead, involves mechanical feedback through stretching and adhesion-deadhesion to the overlying vitelline membrane (Bailles et al., 2019).

### *Drosophila* salivary gland tubulogenesis

Apical-medial myosin-driven apical constriction, or cell wedging, is used again later on in gastrulation during the budding morphogenesis of the tubes of the *Drosophila* salivary glands (Fig. 4C). Here, a combination of apical constriction and directed cell intercalations drives the internalisation of about 100 cells on each side of the ventral midline to form a narrow lumen tube (Booth et al., 2014; Girdler and Röper, 2014; Sanchez-Corrales et al., 2018). This process is under transcriptional control, with the homeotic transcription factor Sex combs reduced defining tissue identity, and a second layer of transcriptional control involving Fork head (Fkh) and Hkb upstream of morphogenetic changes (Fig. 4C′) (Panzer et al., 1992; Andrew et al., 1994; Myat and Andrew, 2000a,b; Myat and Andrew, 2002; Sanchez-Corrales et al., 2021). Both Fkh and Hkb are also upstream of the expression and autocrine secretion of Fog, which, as in the mesoderm and posterior endoderm, activates the cascade that leads to myosin activation (Chung et al., 2017; Sanchez-Corrales et al., 2021). The radial expansion of Fkh and Hkb across the salivary gland placode primordium leads to radial expansion of Fog and to myosin activation. This leads to more and more cells internalising, while intercalations feed more cells towards the invagination point (Sanchez-Corrales et al., 2021). As observed in the mesoderm, in the salivary gland placode, the apical-medial actomyosin network in constricting cells is also tightly coupled across cell-cell junctions into a larger-scale assembly (Sanchez-Corrales et al., 2021).

### *Drosophila* dorsal closure

Around the same time that pulsatile apical area fluctuations and correlated myosin pulsation were first described in mesoderm invagination in the fly embryo, they were also noted within amnioserosa cells during the process of dorsal closure that occurs several hours later in embryogenesis (Fig. 4D; Solon et al., 2009; Blanchard et al., 2010; David et al., 2010). During dorsal closure, as already discussed above, the embryonic epidermis advances on both sides of the dorsal midline to cover the extraembryonic tissue of the amnioserosa. Amnioserosa cells at this point are flat and almost squamous, with vastly larger apical areas than the encroaching epidermal cells, and they display sweeping flows and pulsations of myosin and F-actin (Solon et al., 2009). In contrast with all other processes discussed above, the amnioserosa cells constrict to shrink the overall area covered by the amnioserosa, but their apical area shrinkage is not linked to tissue invagination (Blanchard et al., 2010). The upstream activation of myosin contractility again involves Rho and Rok (Blanchard et al., 2010), though not Fog, which seems to be exclusive to invaginating tissues (Manning and Rogers, 2014). Interestingly, the upregulation of non-muscle myosin II (*zipper*) in the amnioserosa is regulated by an unusual combination of ecdysone and JNK signalling, with the ecdysone receptor EcR binding and working with JNK-activated AP-1 to control expression of a set of genes important for dorsal closure (Fig. 4D′) (Yoo et al., 2021).

### *C. elegans* endoderm invagination

The internalisation of the endodermal precursors that marks the beginning of gastrulation in *C. elegans* is likely the tissue invagination process involving the fewest cells (only two) and therefore involving the smallest ‘supracellular’ actomyosin network (Fig. 4E). However, the similarity with the above internalisation processes suggests that the module of apical constriction and cell wedging, driven by a pulsatile apical-medial actomyosin network connected to cell-cell junctions to drive apical area reduction, is conserved across evolution. The two endodermal cells display flows of actomyosin across the surface that drive apical area shrinkage, though only after a clutch mechanism has been engaged that couples the myosin fluctuations to an inward-moving plasma membrane (Fig. 4E′) (Roh-Johnson et al., 2012). This is very similar to the *Drosophila* mesoderm, where initially unproductive apical area fluctuations are also transformed into productive, shrinkage-inducing fluctuations (Roh-Johnson et al., 2012).

### Mouse intestinal crypt formation

In vertebrates, apical constriction involving apical accumulation of myosin is also a key driver of morphogenetic changes such as folding of epithelial tissues (Haigo et al., 2003; Lee and Harland, 2007). Whether the same dynamic pools of apical-medial myosin are involved is not yet clear. In the process of crypt morphogenesis of the mammalian intestine, there is evidence to suggest that an apical-medial myosin-mediated mechanism is at play (Fig. 4F) (Sumigray et al., 2018)). Crypts form from pre-existing intervillar regions in the intestine in the first 10 days after birth. Crypt progenitor cells begin to apically constrict to drive a further narrower invagination of the intervillar region, and labelling of active phosphorylated myosin shows strong apical-medial pools in these cells that are lacking prior to the onset of apical constriction. Transcriptome analysis revealed upregulation of non-muscle myosin genes (*Myh9* and *Myl9*) as well as of a Rho-GEF (*Ect2*) and actin nucleators (*Diaph2* and *Diaph3*) (Fig. 4F′), and MyoIIA/C double knockout intestines show a strong reduction of apical constriction of the crypt precursors (Sumigray et al., 2018).

## Common regulatory themes for supracellular apical-medial networks and emergent properties

The above examples of interlinked supracellular apical-medial actomyosin networks of varying sizes driving apical area reduction display many common themes, strongly suggesting that this is a very common and well-conserved mechanism of tissue morphogenesis across evolution. Some common control and regulatory themes have emerged, including apical-medial actomyosin pulses, which are cycles of increases and decreases in intensity, as well as flows of the strongest actomyosin foci across the apical surface. These actomyosin pulses can generate both unproductive and productive apical area changes, i.e. leading to either area fluctuations or apical constriction, respectively (Martin et al., 2009; Roh-Johnson et al., 2012; Booth et al., 2014). To turn unproductive pulses into productive pulses, in some systems it seems a molecular clutch becomes engaged that allows stabilisation of a shrunken apical surface area. During mesoderm invagination in *Drosophila,* the pulsation and clutch are established under the control of separate transcription factors, with Snail controlling the constriction and Twist controlling the following stabilisation of shape (Fig. 5A) (Martin et al., 2009, 2010). In *C. elegans* endodermal cell internalisation, engagement of the clutch is dependent both on cadherin as well as the Rac GTPase (Roh-Johnson et al., 2012). A further factor influencing whether actomyosin fluctuations are productive or not appears to be the duration of pulses: in each system, pulse duration appears to have a specific range over which it elicits productive shrinkage. If the cycle length is much longer than this range, fluctuations become unproductive (Gorfinkiel and Blanchard, 2011; Booth et al., 2014).

Studies in the *Drosophila* mesoderm and amnioserosa have shed light on the arrangement of actin and myosin in the medial networks, as well as their regulatory control. Within the mesoderm and likely also in other tissues, Rho activation at the cortex and in the apical-medial domain activates different downstream effectors: the actin regulator Diaphanous (Dia) at the cortex and Rok in the apical-medial region (Fig. 5B) (Mason et al., 2013). Consistent with this, F-actin in individual mesodermal cells within the apical domain is organised in a radial polarised arrangement, with barbed ends where new monomers are incorporated at the cortex, and pointed ends enriched in the medial domain where myosin is accumulated (Fig. 5C) (Coravos and Martin, 2016). This arrangement is reminiscent of sarcomeres. Furthermore, as observed in the amnioserosa and germband cells, the apical F-actin network comes in two flavours: a persistent network that is independent of active Rho but dependent on the formin Frl; and a pulsatile network that requires activation by Rho-GTP (Fig. 5D) (Dehapiot et al., 2020).

Interestingly, in several of the systems introduced above, the apical-medial actomyosin depends crucially on the underlying microtubule cytoskeleton for its function in mediating successful constriction (Fig. 5E,F) (Booth et al., 2014; Ko et al., 2019; Guru et al., 2022). In salivary gland placodal cells, the microtubule cytoskeleton in the cells that are about to apically constrict undergoes a regulated rearrangement into a non-centrosomal longitudinal array (Fig. 5E) (Booth et al., 2014). Microtubule minus ends are anchored by Patronin within the apical domain, in very close physical apposition to the apical-medial actomyosin (Gillard et al., 2021). If microtubules are experimentally eliminated through severing, or if the longitudinal array is prevented from forming, then the amount of apical-medial actomyosin and apical constriction is reduced (Booth et al., 2014; Gillard et al., 2021). Microtubules also play an important role in mesoderm invagination in the fly, though in a variation of the theme (Fig. 5F). Here, non-centrosomal microtubules seem to promote connection between apical-medial actomyosin and adherens junctions, to assist repair of such connections, suggesting a more indirect role in apical constriction (Ko et al., 2019). In yet another variant, short, non-centrosomal apical microtubules in amnioserosa cells, which are dependent on microtubule-binding proteins EB1 and Patronin, support the coalescence of apical pulses (Guru et al., 2022).

The overall close apposition of apical actomyosin pools that drive apical constriction, and microtubules (in particular longitudinal microtubule bundles) appears to be conserved across evolution. During gastrulation in *Xenopus*, longitudinal microtubule bundles also terminate apically in invaginating bottle cells, near where myosin is concentrated apically, and microtubule disruption, but not stabilisation, leads to loss of apical constriction (Lee and Harland, 2007).

Actomyosin networks are intrinsically mechanosensitive (Etienne et al., 2015) and therefore the actomyosin in individual cells within a network is likely influenced by the contractility and behaviour of actomyosin assemblies in its neighbours. For apical-medial actomyosin networks, such mechano-feedback could lead to strengthening of contractile events, to in-phase or out-of-phase coupling of contractile cycles in neighbours, or to the spread of contractile behaviour across a tissue. There is evidence for at least some of these effects, such as out-of-phase coupling in the *Drosophila* mesoderm (Martin et al., 2009) and in-phase-coupling in the *Drosophila* amnioserosa (Solon et al., 2009).

The apical-medial network in the mesoderm during *Drosophila* gastrulation shows structural redundancy in its connectivity, thereby ensuring robustness of the network even when functional nodes are missing (Yevick et al., 2019). Whether this structural robustness and redundancy is the only functional result of the apparent supracellularity of this network remains to be determined. Recent work illustrates that the porosity of the actomyosin network within the myosin activation cascade in the mesoderm is controlled by the Gα_12/13_ protein Concertina, but not through the RhoGEF2-binding transmembrane protein T48 (Horo et al., 2024).

## Summary and outlook

Supracellular actomyosin assemblies are intriguing, as they have the unique property of physically bridging the individual cell scale to the tissue scale, thereby allowing tissue-scale coordination and feedback, whilst components and regulators of the assemblies are encoded in the transcriptional blueprint of each individual cell. Their common functions are reiteratively used in many morphogenetic processes during development, and we will likely discover many more such instances across different animals. Moving forward, the identification and analysis of emergent properties that depend on the supracellular quality of these assemblies will be an exciting avenue for research. In particular, it will be important to discover whether any such properties or functions require a minimum size of the assembly and whether assemblies are supported by specific constituents or work at different time scales – all aspects that could determine how supracellular assemblies function as molecular machines throughout morphogenesis.
